# Does wearing clothing made of a synthetic “cooling” fabric improve indoor cycle exercise endurance in trained athletes?

**DOI:** 10.14814/phy2.12505

**Published:** 2015-08-19

**Authors:** Sara J Abdallah, Robin Krug, Dennis Jensen

**Affiliations:** 1 Clinical Exercise & Respiratory Physiology Laboratory, Department of Kinesiology & Physical Education, McGill UniversityMontréal, QC, Canada; 2 McGill Research Centre for Physical Activity and Health, McGill UniversityMontréal, QC, Canada; 3 Respiratory Epidemiology & Clinical Research Unit, Division of Respiratory Medicine, Department of Medicine, McGill University and the Research Institute of the McGill University Health CenterMontréal, QC, Canada

**Keywords:** Cycling, performance, thermoregulation, V˙o^2max^

## Abstract

This randomized, double-blind, crossover study examined the effects of a clothing ensemble made of a synthetic fabric promoted as having superior cooling properties (COOL) on exercise performance and its physiological and perceptual determinants during cycle exercise in ambient laboratory conditions that mimic environmental conditions of indoor training/sporting facilities. Twenty athletes (15 men:5 women) aged 25.8 ± 1.2 years (mean ± SEM) with a maximal rate of O_2_ consumption of 63.7 ± 1.5 mL·kg^−1^·min^−1^ completed cycle exercise testing at 85% of their maximal incremental power output to exhaustion while wearing an ensemble consisting of a fitted long-sleeved shirt and full trousers made of either COOL or a synthetic control fabric (CTRL). Exercise endurance time was not different under COOL versus CTRL conditions: 12.38 ± 0.98 versus 11.75 ± 1.10 min, respectively (*P* > 0.05). Similarly, COOL had no effect on detailed thermoregulatory (skin and esophageal temperatures), cardiometabolic, ventilatory, and perceptual responses to exercise (all *P* > 0.05). In conclusion, clothing made of a synthetic fabric with purported “cooling” properties did not improve high-intensity cycle exercise endurance in trained athletes under ambient laboratory conditions that mimic the environmental conditions of indoor training/sporting facilities.

## Introduction

Maintenance of a stable core body temperature (or rate of body heat storage equivalent to zero) involves the delicate balance between the net rates of body heat production and body heat dissipation by conduction, convection, radiation, evaporation, and respiration (Gavin 2003; Kenny and Jay 2013). During exercise, increased skeletal muscle metabolic demands are associated with progressive and intensity-dependent increases in body heat production, which must be dissipated to prevent excessive increases in the rate of body heat storage and, by extension, core and muscle tissue temperatures (Ingram and Mount 1975; Kenny and Jay 2013). In humans, evaporation of sweat from the skin surface accounts for ∼80% of heat loss during physical activity and is thus the primary means of thermoregulation during exercise (Gavin 2003; Kenney et al. 2012). As such, any disturbance to evaporative heat loss from the skin surface, especially during strenuous exercise, has the potential to compromise the net rate of heat loss to the environment and increase the rate of body heat storage with attendant compromisation of thermoregulatory balance (Aoyagi et al. 1998) and exercise performance.

Clothing represents a layer of insulation that forms a barrier to evaporative heat loss by compromising heat dissipation from the skin surface, ultimately decreasing cooling efficiency and increasing core body temperature, skin temperature (*T*_skin_) and subjective ratings of skin wettedness and body temperature, particularly during exercise (Berglund and Gonzalez 1977; Nagata 1978; Pascoe et al. 1994; Gavin 2003; Davis and Bishop 2013). It follows that clothing fabrics that impose the least amount of resistance to evaporation (i.e., high vapor permeability, low thermal insulation) have the potential to optimize thermoregulatory balance (i.e., maintain the rate of heat storage near zero) during physical activity (Gavin 2003) and enhance exercise performance.

Clothing and textile manufacturers have created synthetic fabrics with enhanced evaporative characteristics that have been marketed to consumers as having superior heat loss (or “cooling”) properties capable of improving human exercise performance by enhancing physiological (e.g., thermoregulatory) responses to exercise (Bishop et al. 2013; Davis and Bishop 2013). These claims have been largely unfounded, with the majority of published studies indicating a lack of thermoregulatory advantage during exercise while wearing clothing made of synthetic versus natural (e.g., cotton) fabrics (Bishop et al. 2013; Davis and Bishop 2013). However, the majority of these studies was performed during mild-to-moderate intensity exercise (30–70% of maximal rate of O_2_ consumption; 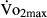) of fixed and variable durations (10–60 min) in relatively small groups (*n* ≤ 11) of healthy trained (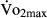 ≥ 55 mL·kg^−1^·min^−1^) and untrained/recreationally active adults (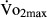 < 55 mL·kg^−1^·min^−1^) (Gavin et al. 2001; Dai et al. 2008; Brazaitis et al. 2010; Corbett et al. 2015; De Sousa et al. 2014). Furthermore, these studies were designed to unveil the cooling properties of synthetic fabrics worn during outdoor sporting activities and were thus carried out in environmental chambers with controlled temperature, humidity, and wind speeds. However, given the recent indoorization of outdoor sports (van Bottenburg and Salome 2010) and the use of wind-free, humidity controlled, and temperate (∼20–23°C) indoor training/sporting facilities, clothing made of synthetic cooling fabrics will invariably be worn by athletes competing and/or training indoors, where (1) sweat rates as high as 1.8 L·h^−1^ can be achieved (Hamouti et al. 2010); and (2) sweat rate and cooling efficiency becomes largely dependent on exercise intensity and duration, cardiorespiratory fitness (Sawka and Young 2006) and clothing; and largely independent of ambient temperature, wind velocity and relative humidity. As such, wearing clothing made of a synthetic fabric with enhanced cooling properties may be especially advantageous during strenuous exercise performed indoors by trained athletes.

Accordingly, the purpose of this randomized, double-blind, crossover study was to test the hypothesis that wearing a clothing ensemble made of a synthetic fabric promoted by the manufacturer as having superior cooling properties would improve exercise performance of endurance trained athletes under ambient laboratory conditions that mimic the temperate environmental conditions of indoor training/sporting facilities. We further hypothesized that this improvement in exercise performance would be associated with improvements in thermoregulatory, cardiometabolic, ventilatory, and perceptual responses to exercise.

## Materials & Methods

### Participants

Participants included nonsmoking men and women aged 18–40 years with normal spirometry and a 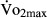 on incremental cycle exercise testing of ≥60 mL·kg^−1^·min^−1^ and/or ≥125% of the predicted maximum value (Jones et al. 1985). Participants were recruited from the McGill University Olympic, Cycling, Running and Triathlon clubs as well as through contact with coaches of competitive cycling and triathlon teams/training groups in the Montréal and surrounding area. Participants were excluded if they had a known or suspected history of cardiovascular, respiratory, metabolic, musculoskeletal, endocrine and/or neuromuscular disease; were taking doctor prescribed medications, other than oral contraceptives. Eumenorrheic women not taking oral contraceptives were studied during the luteal phase of their menstrual cycle (assumed to be 14 days for all women [Chabbert Buffet et al. 1998]), which was determined using the first day of the last menstrual cycle and the average length of at least three previous cycles.

### Study design

This was a controlled, randomized, double blind, crossover study wherein participants visited the laboratory on three separate occasions over a period of 2 weeks. *Visit 1* included spirometry and an incremental cycle exercise test to determine maximal power output (MPO). *Visits 2* and *3* included spirometry followed by a constant work rate cycle exercise test at 85% MPO with added measurement of *T*_skin_ and esophageal temperature (*T*_eso_) under one of two conditions, randomized to order: wearing a garment made of a synthetic control fabric (CTRL) or a fabric with purported superior cooling properties (COOL).

Prior to exercise at *Visits 2 and 3*, participants were fitted with *T*_eso_ and *T*_skin_ probes and then lay supine for ≥30-min at rest while baseline measures of *T*_eso_ and *T*_skin_ were collected. Exercise testing commenced once baseline measures of *T*_eso_ varied by ≤0.05°C for ≥10 min. Immediately before and after exercise tests, the participant’s nude and dry body mass was recorded to the nearest 2 g (KCC 150, Mettler-Toledo Inc., Mississauga, ON, Canada), while the mass of all clothing (except shoes) were recorded to the nearest 0.01 g (ALD 4102; Dini Argeo, Modena, Italy). Clothing ensembles were weighed in a sealed plastic bag to avoid evaporative water loss. Preexercise body mass was adjusted for the volume (mass) of water consumed during insertion of the *T*_eso_ probe.

Experiments were conducted under ambient laboratory conditions where temperature and relative humidity (RH) were maintained within the recommended temperature range set by the American College of Sports Medicine (ACSM’s 2012) for indoor facilities under both CTRL and COOL conditions: 24.4 ± 0.2 versus 24.5 ± 0.2°C (*P* = 0.79); and 23.7 ± 8.9 versus 22.5 ± 8.4% RH (*P* = 0.23). Participants were instructed to avoid alcohol, caffeine, and strenuous exercise on each test day. Fans were not used to simulate wind nor was fluid intake permitted during the preexercise baseline periods or exercise tests. All visits were separated by ≥48 h and were conducted at the same time of day (±1 h) for each participant. The study protocol and consent form were approved by the Institutional Review Board of the Faculty of Medicine at McGill University (A10-M108-13A) in accordance with the *Declaration of Helsinki*. All participants provided written informed consent.

### Clothing ensembles and randomization

Two prewashed clothing ensembles, both of warp knit single-layer construction and consisting of a fitted long-sleeved shirt, full trousers and underwear, were examined: one made of a synthetic control fabric (CTRL; 80% nylon:20% spandex; 195 gr·m^−2^; nylon 40 denier/10 filaments + spandex 40 denier) and the other made of a synthetic fabric claimed by the manufacturer and study sponsor (Lamour Hosiery Inc., Montréal, QC, Canada) to have superior cooling properties and evaporative characteristics (COOL; 78% nylon:22% spandex; 160 gr·m^−2^; nylon 35 denier/24 filaments + spandex 30 denier). A third party statistician completely uninvolved with the study prepared the randomization schedule. Prior to the start of the study, the manufacturer and study sponsor provided the investigators with the clothing ensembles in boxes labeled “Ensemble A” and “Ensemble B”.

### Spirometry

Forced expiratory volume in 1-s (FEV_1_), forced vital capacity (FVC) and the FEV_1_/FVC ratio were determined with participants seated, utilizing recommended techniques (Miller et al. 2005) and automated equipment (Vmax Encore 29C; CareFusion, Yorba Linda, CA, USA).

### Exercise testing

Exercise tests were conducted on an electronically braked Velotron Pro cycle ergometer using Version 1.6 of the Velotron Coaching Software (RacerMate Inc.; Seattle, WA, USA). Incremental exercise tests consisted of a baseline resting period of ≥6-min, followed by 25 watt increases in power output every 2-min (starting at 150 watts for men and 100 watts for women): MPO was defined as the highest power output the participant was able to sustain for ≥30-s. Constant power output exercise tests consisted of a baseline resting period of ≥6-min, followed by a 2-min warm-up at 25% MPO and then a step increase in work rate to 85% MPO (rounded up to the nearest watt): exercise endurance time (EET) was defined as the duration of loaded pedaling, including the 2-min warm-up. Participants remained seated and maintained a pedaling cadence of 50–95 rev·min^−1^ throughout each test. Exercise tests were terminated at volitional fatigue (exhaustion) or once pedaling cadence dropped below 50 rev·min^−1^, whichever occurred first.

Standard cardiometabolic and gas exchange parameters were collected breath-by-breath at rest and during exercise (Vmax Encore 29C) while participants breathed through a mouthpiece and a low-resistance flow transducer with nasal passages occluded by a noseclip. Heart rate (HR) was monitored continuously by 12-lead ECG.

### Skin and esophageal temperature

Skin temperatures were recorded using temperature probes (SST-1; Physitemp Instruments Inc., Clifton, NJ, USA) secured with adhesive dressings (Tegaderm; 3M Health Care, Neuss, Germany) at the following sites: center of the forehead (*T*_forhead_); hand at the right middle finger (*T*_finger_); right forearm (*T*_forearm_); chest at the right pectoralis (*T*_chest_); middle of the right vastus lateralis (*T*_thigh_); and back at the right latissimus dorsi (*T*_back_). *T*_eso_ was measured using an esophageal temperature probe (ESO-1; Physitemp Instruments Inc.) and used as an index of core body temperature. After “numbing” of the nasal and pharyngeal passages with a 2% endotracheal lidocaine spray (Lidodan^TM^; Odan Laboratories Ltd., Montréal, QC, Canada), the *T*_eso_ probe was passed through the nose and positioned in the esophagus at a depth of one quarter of the participant’s standing height. All temperatures were sampled at 60 Hz and digitized using the Thermes USB temperature data acquisition system in conjunction with the DASYLab Basic software (Physitemp Instruments Inc.). Before each test, the Thermes USB and temperature probes were calibrated in accordance with the manufacturer’s instructions.

Mean *T*_skin_ was calculated using a modification of the Hardy et al. (1938) equation: (0.05 × *T*_finger_) + (0.14 × *T*_forearm_) + (0.07 × *T*_head_) + (0.19 × *T*_thigh_) + [0.175 × (*T*_chest_ + *T*_back_)]. Mean body temperature (*T*_body_) was calculated as: (0.65 × *T*_eso_) + (0.35 × *T*_skin_). Heat storage (HS; kcal·m^−2^·h^−1^) was calculated as: {0.83 × [body mass (kg)/body surface area (m^2^)] × (Δ*T*_eso_/Δt)}, where the constant of 0.83 is the specific heat of the body, Δ*T*_eso_ is the exercise-induced change in *T*_eso_ in °C, and Δt is the EET in hours. Metabolic energy production (MEP, W·m^−2^) was estimated using the following equation: {[(0.23 × RER_max_) + 0.77] × 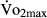 × K × 60/body surface area (m^2^)}, where RER_max_ is the respiratory exchange ratio at maximal exercise and K is the energy equivalent of O_2_ (5.873 W·h^−1^·L^−1^·min^−1^) (Gavin et al. 2001). Mass of sweat evaporated (S_WE_, g) from the participants during exercise was determined from the exercise-induced change in body mass (ΔBM) using the following equations: ΔBM − RES_LOSS_ − MET_LOSS_ − S_WNE_, where RES_LOSS_ (g·min^−1^) is the water loss attributed to respiration {calculated as ([0.173 × MEP × (5.87 − P_a_) × body surface area (m^2^) × 60]/2408), where P_a_ is the ambient water vapor pressure in pascals} and MET_LOSS_ (g·min^−1^) is the metabolic mass loss (calculated as [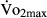 × 44 × (RER_max_ − 32)], where and S_WNE_ is the mass (g) of nonevaporated sweat calculated as the difference in clothing mass measured before and immediately after exercise) (Gavin et al. 2001). Sweat efficiency (%) was calculated as S_WE_/exercise-induced change in body mass (ΔBM, g), while sweat rate (L·h^−1^) was calculated as ΔBM/EET (Gavin et al. 2001).

### Perceptual responses

At rest, within the last 30-s of every 2nd min during exercise and at end-exercise, participants provided ratings of: clothing comfort using a 7-point scale (+3, very comfortable; +2 comfortable; +1, slightly comfortable; 0 neutral; −1 slightly uncomfortable; −2, uncomfortable; −3, very uncomfortable); thermal sensation using a 9-point scale (−4, very hot; −3, hot; −2, warm; −1 slightly warm; 0, neutral; +1, slightly cool; +2, cool; +3, cold; +4, very cold); skin wettedness using a 7-point scale (−3, too wet; −2, wet; −1, slightly wet; 0, neutral; +1, slightly dry; +2, dry; +3, too dry); and of breathing and leg discomfort using Borg’s 0–10 category ratio scale (Borg 1982). At end-exercise, participants verbalized their main reason(s) for stopping (breathing discomfort; leg discomfort; thermal strain, i.e., “too hot to continue”; or fatigue); and to quantify the percentage contribution of breathing discomfort, leg discomfort, thermal strain and fatigue to exercise cessation.

### Debriefing

Upon completion of all procedures at *Visit 3*, participants identified which visit (2 or 3) they found the ensemble: to be more comfortable; to have the best “cooling” effect; and to be best suited for their training. Participants were also asked to identify which visit (2 or 3) they believed the COOL ensemble was worn, and to identify which visit (2 or 3) they believe they achieved their best exercise performance.

### Analysis of exercise end-points

Temperature measurements were averaged over the last 10-min of the preexercise rest (baseline) period and in 15-s intervals during exercise. Physiological parameters measured breath-by-breath were averaged in 30-s intervals at rest and during exercise. Temperature measurements collected over the second 15-s interval of every 2nd minute during exercise were linked with physiological and perceptual parameters, respectively, collected over the first and last 30-s interval of every 2nd minute during exercise. Measured parameters were evaluated at three main time points: (1) *preexercise rest*; (2) *isotime*, defined as the highest equivalent time achieved during each of the constant power output cycle exercise tests performed by a given participant; and (3) peak exercise, defined as the average of the 30-s of loaded pedaling.

### Statistical analysis

The effect of clothing ensemble, measurement time, and their interaction on measured parameters was examined using a two-way repeated-measures analysis of variance with correction for multiple comparisons using Tukey’s honest significant difference test (SigmaStat®; Systat® Software Inc., San Jose, CA). Fisher’s exact test was used to examine the effect of clothing ensemble on the reasons for stopping exercise. Two-tailed paired *t*-tests were used to examine the effect of clothing ensemble on the percentage contribution of breathing discomfort, leg discomfort, thermal strain, and fatigue to exercise cessation. Participant responses to the debriefing questions were assessed by Chi-square. Blinding was opened only after these analyses were completed. Significance was set at *p* < 0.05 and data are presented as means ± SEM.

## Results

### Participant characteristics

Twenty athletes (15 men:5 women) with the following characteristics completed the study: age, 25.8 ± 1.2 years; body mass, 68.1 ± 2.3 kg; body height, 172.5 ± 2.1 cm; body mass index, 22.8 ± 0.4 kg·m^−2^; FEV_1_, 4.37 ± 0.16 L (108 ± 2% predicted [Hankinson et al. 1999]); FEV_1_/FVC, 80.2 ± 1.3%; 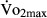, 63.7 ± 1.5 mL·kg^−1^·min^−1^ (range: 56.5–74.1 mL·kg^−1^·min^−1^), and 161 ± 36% predicted (range: 125–225% predicted); and MPO, 317.5 ± 11.2 watts (128 ± 4.9% predicted [Jones et al. 1985]).

### Exercise responses

**Figure 1:**
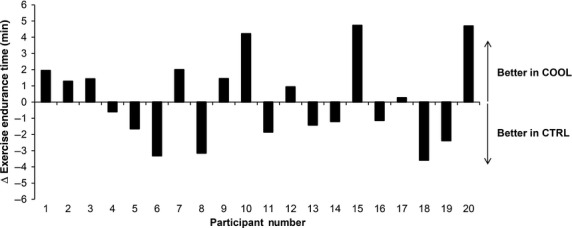
Effects of a cooling fabric (COOL) on exercise endurance time (EET) of each subject. The change (Δ) in EET was calculated as the difference in EET between COOL and control fabric (CTRL) trials.

Intervention order was balanced such that 12 of the 20 participants were randomized to exercise in the COOL ensemble first. There was no difference in EET between the CTRL and COOL conditions: 11.75 ± 1.10 versus 12.38 ± 0.98 min, respectively (*P* = 0.32). In order to rule out a potentially confounding order effect on EET, we compared EET between *Visit* 2 and *Visit* 3, irrespective of clothing ensemble and found no difference: 12.24 ± 0.95 versus 12.39 ± 1.0 min, respectively (*P* = 0.79). As illustrated in Figure1, 10 participants increased their EET (by 2.30 ± 0.56 min), while 10 participants decreased their EET (by 2.03 ± 0.33 min) during COOL versus CTRL exercise.

**Figure 2:**
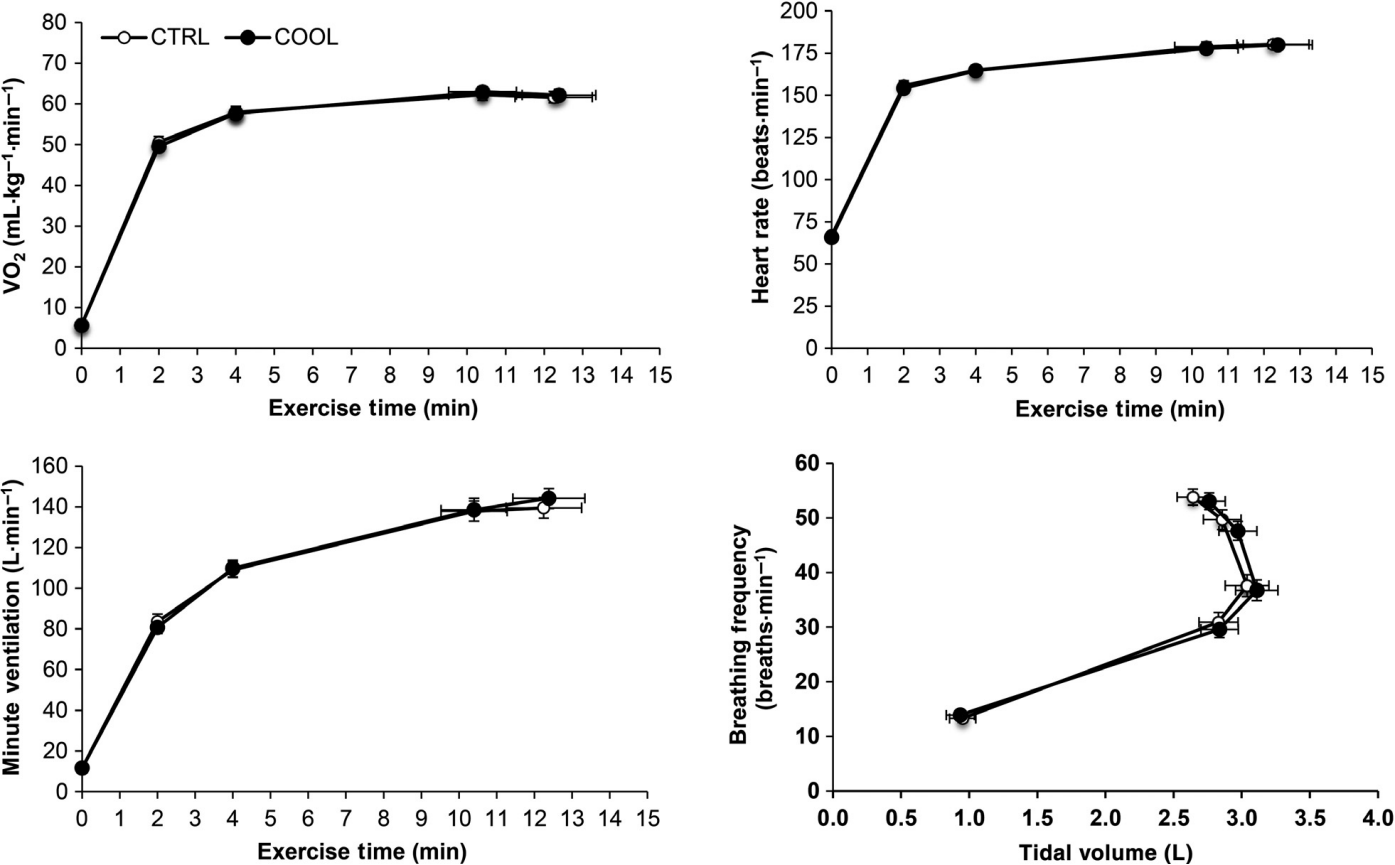
Comparison of temporal changes in cardiopulmonary responses during cycle exercise testing at 85% of maximal incremental power output (equivalent to 267 ± 9 watts) while wearing control (CTRL) or cooling (COOL) clothing ensembles. Data points are means ± SEM at rest, at standardized submaximal exercise times (including isotime, equivalent to 10.4 ± 0.9 min) and peak exercise. 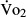, rate of oxygen consumption.

**Figure 3:**
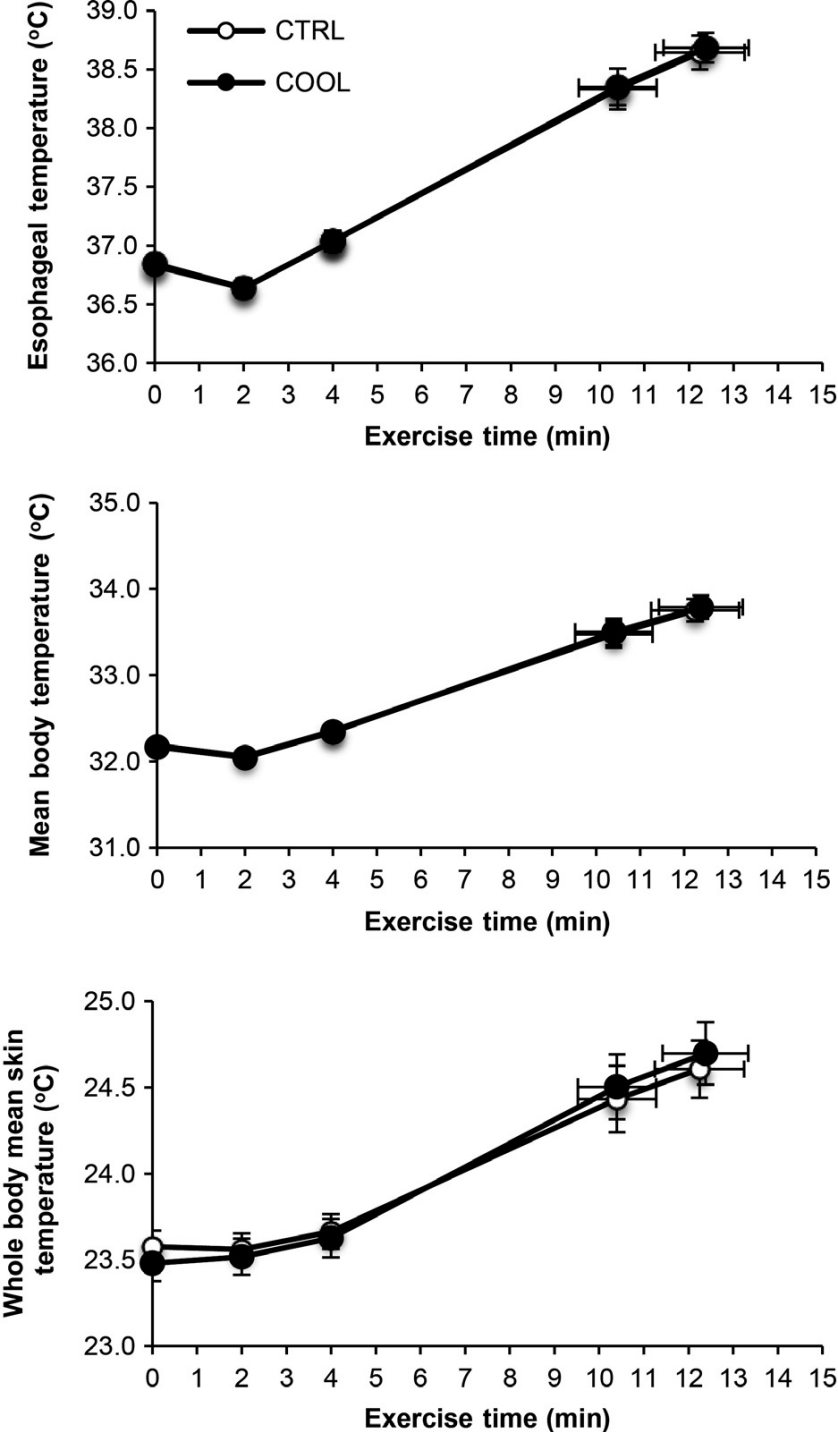
Comparison of temporal changes in esophageal temperature, mean body temperature, and whole-body mean skin temperature between control (CTRL) and cooling (COOL) ensembles worn during cycle exercise testing at 85% of maximal incremental power output (equivalent to 267 ± 9 watts). Data points are means ± SEM at rest, at standardized submaximal exercise times (including isotime, equivalent to 10.4 ± 0.9 min) and peak exercise.

Cardiometabolic and ventilatory responses to exercise were not different under COOL versus CTRL conditions (Fig.2). There was also no difference in *T*_forhead_, *T*_finger_, *T*_forearm_, *T*_chest_, *T*_thigh_*,* and *T*_back_ between COOL and CTRL conditions at rest or at any point during exercise. *T*_eso_, mean body and whole body mean skin temperature increased progressively from rest to end-exercise, with no differences between COOL and CTRL conditions (Fig.3). Similarly, there was no difference between COOL and CTRL for each of the following: HS, 287.4 ± 9.8 versus 307.9 ± 20.1 kcal·m^−2^·h^−1^ (*P* = 0.23); sweat efficiency, 88 ± 0.01 versus 87 ± 0.01% (*P* = 0.47); sweat rate, 1.84 ± 0.09 versus 1.98 ± 0.19 L·h^−1^ (*P* = 0.35); exercise-induced increase in garment mass (54.42 ± 11.03 vs. 56.81 ± 13.00 g; *P* = 0.65) and decrease in body mass (385.1 ± 40.1 vs. 378.3 ± 48.1 g; *P* = 0.79).

### Perceptual and debriefing responses

**Figure 4:**
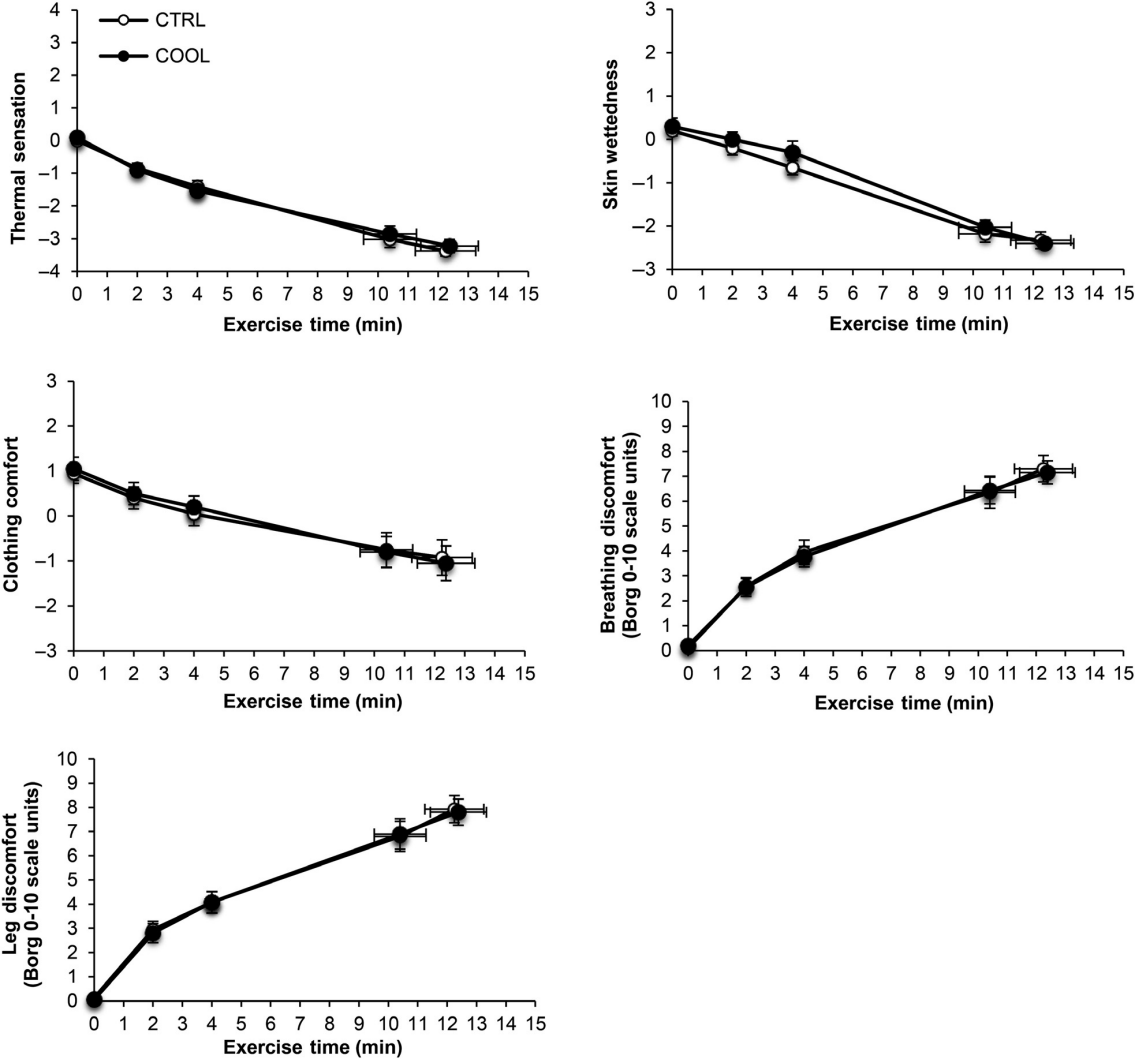
Comparison of temporal changes in thermal sensation, skin wettedness, clothing comfort, breathing discomfort, and leg discomfort during cycle exercise at 85% of maximal incremental power output (equivalent to 267 ± 9 watts) while wearing control (CTRL) or cooling (COOL) clothing ensembles. Data points are means ± SEM at rest, at standardized submaximal exercise times (including isotime, equivalent to 10.4 ± 0.9 min) and peak exercise.

Subjective ratings of thermal sensation, skin wettedness, clothing comfort, and breathing and leg discomfort were not different at any time during exercise under COOL versus CTRL conditions (all *P* > 0.05; Fig.4). The reasons for stopping exercise were also similar between trials: leg discomfort (COOL, 70% vs. CTRL, 85% of respondents; *P* = 0.451); breathing discomfort (COOL, 15% vs. CTRL, 5% of respondents; *P* = 0.605); fatigue (COOL, 10% vs. CTRL, 5% of respondents; *P* = 1.0); and thermal strain (COOL, 5% vs. CTRL, 5% of respondents; *P* = 1.0). The relative contribution of leg discomfort (COOL, 56.3 ± 8.2% vs. CTRL, 62.5 ± 6.6%; *P* = 0.471), breathing discomfort (COOL, 24.5 ± 6.1% vs. CTRL, 23.5 ± 4.4%; *P* = 0.841), fatigue (COOL, 8.5 ± 5.3% vs. CTRL, 4.3 ± 4.0%; *P* = 0.539), and thermal strain (COOL, 7.0 ± 4.4% vs. CTRL, 4.3 ± 2.8%; *P* = 0.584) to exercise cessation were also similar between COOL and CTRL conditions.

**Figure 5:**
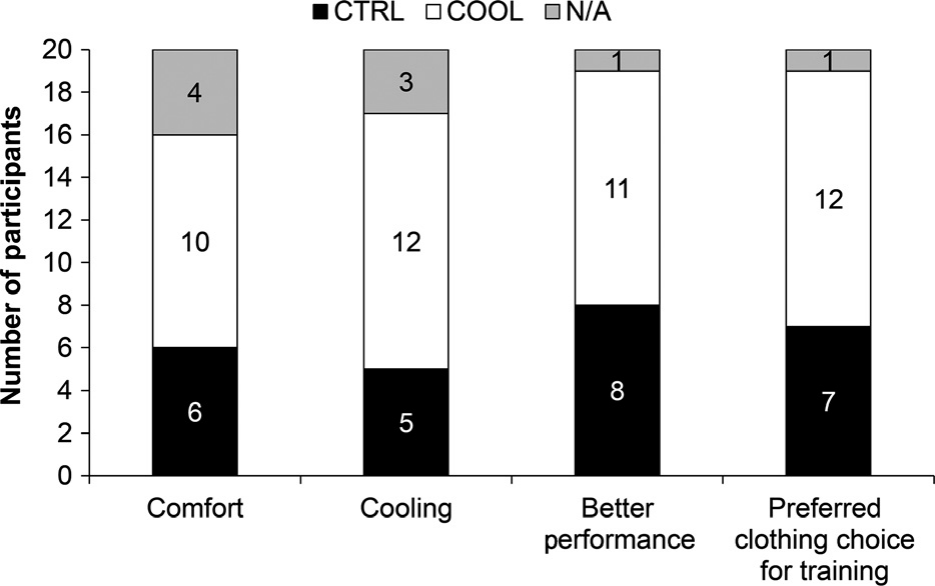
Participant feedback on perceived clothing comfort, cooling effect, enhanced performance and clothing choice for training between the control (CTRL) and cooling (COOL) ensembles. The number of participants that reported a preference for CTRL, COOL, or no preference (N/A) is presented.

Although no statistically significant differences between COOL and CTRL on debriefing responses were identified, a higher mean percentage of our participants favored exercising in the COOL versus CTRL ensemble, with 50% (vs. 30%) and 60% (vs. 25%) identifying the former as feeling more comfortable and as having more of a “cooling” effect, respectively (Fig.5). Furthermore, 55% (vs. 40%) of participants believed they performed best while wearing the COOL ensemble (73% of whom increased their EET under COOL vs. CTRL conditions by 2.4 ± 0.7 min); however, only 15% of these individuals attributed their perceived enhanced performance to the COOL ensemble. Finally, 60% (vs. 35%) of participants identified the COOL ensemble as the one they would prefer to wear during training, with all of these individuals citing the “lighter, cooler fabric” as the reason for their choice.

## Discussion

In contrast to our a priori hypotheses, the results of this study in trained athletes indicate that wearing a clothing ensemble made of a synthetic fabric with purported superior “cooling” properties did not improve high-intensity exercise performance nor was it associated with improved physiological and/or perceptual responses to exercise performed under laboratory conditions that mimicked the temperate environments of indoor training/sporting facilities.

Previous studies examining the effects of synthetic fabrics on thermoregulatory responses during mild-to-moderate intensity exercise (30–70% of 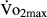) in untrained and/or recreationally active adults may not accurately reflect the cooling efficiency of these fabrics in as much as: mild-to-moderate intensity exercise does not impose a physiological strain great enough to significantly alter core body temperature and thermoregulatory balance (Bishop et al. 2013; Davis and Bishop 2013); and untrained and/or recreationally active adults with normal cardiorespiratory fitness levels may not be able to achieve and/or sustain the power outputs and metabolic rates needed to impose a meaningful physiological heat stress on the body. Furthermore, individuals should be fully clothed in order to maximize the surface area of the skin covered by the test fabric as well as minimize heat loss occurring independent of the test fabric. That is, the clothing ensemble should not be limited to a t-shirt and/or shorts, as has been the case in most previous studies. To circumvent the methodological limitations of earlier studies, we (1) studied endurance trained men and women capable of achieving abnormally high maximal power outputs and metabolic rates; (2) employed a constant work rate cycle endurance exercise test at 85% MPO*,* equivalent to 267 ± 9 watts; and (3) minimized the surface area for evaporative heat loss by clothing our participants in a fitted long-sleeved shirt and full trousers. Indeed, *T*_eso_ increased progressively from ∼36.8°C at rest to ∼38.7°C at end-exercise under both COOL and CTRL conditions, with six athletes achieving a maximum *T*_eso_ of >39.0°C under both experimental conditions. These peak core body temperatures are higher than those reported by most studies that employed mild-to-moderate exercise intensities (<60% 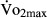; [De Sousa et al. 2014; Ha et al. 1999; Kwon et al. 1998]), but comparable to those reported by investigators who utilized higher intensity exercise testing protocols (>60% 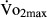) in a hot environment of >26°C (Gonzalez-Alonso et al. 1999; Brazaitis et al. 2010; Sperlich et al. 2013). Furthermore, the sweat rates of 1.84–1.98 L·h^−1^ achieved during exercise in our study, although not different between COOL and CTRL, were considerably higher than those previously reported in trained and untrained individuals during mild-to-moderate exercise intensities in a hot environment of >30°C (Mora-Rodriguez et al. 2010), yet comparable to those reported during high-intensity exercise (Hamouti et al. 2010; Mora-Rodriguez et al. 2010).

Under the experimental conditions of our study (i.e., temperate indoor environment), the COOL ensemble did not improve EET nor was it associated with alterations in thermoregulatory, cardiometabolic, and/or ventilatory responses to exercise. These findings are consistent with Gavin et al. (2001) who found no effect of a synthetic versus natural (100% cotton) fabric on *T*_body_, *T*_skin_, metabolic, and perceptual responses during 30-min of treadmill running at 70% of 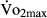 in a hot (30°C) and humid (35% RH) environment with a simulated wind of 11 km·h^−1^ in 8 trained athletes. Similarly, a study of eight recreationally active men by Brazaitis et al. (2010) observed no effect of wearing t-shirt made of 93% polyester versus 94% cotton on thermoregulatory, physiological, and perceptual responses during treadmill running at 8 km·h^−1^ and 1° grade. A study of eight endurance trained runners by Sperlich et al. (2013) also reported that t-shirts made of 90–100% polyester (vs. 100% cotton) were not associated with improved time to exhaustion, *T*_body_, *T*_skin_, cardiometabolic, and ventilatory responses during submaximal and high-intensity treadmill running under hot (31.7°C) and humid (42% RH) environmental conditions. Finally, a study of 10 recreationally active men by De Sousa et al. (2014) recently reported no effect of wearing a t-shirt made of synthetic fabrics (81% polyester:19% elastin) versus 100% cotton on *T*_skin_, cardiometabolic and perceptual responses to 45 min of cycle exercise at 50% of 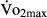 in a hot (33°C) and humid (60% RH) environment.

The COOL and CTRL fabrics studied herein were not sufficiently distinct to provoke a change in subjective ratings of thermal sensation, skin wettedness, clothing comfort, breathing, and/or leg discomfort during exercise. These findings are consistent with previous studies reporting no effect of synthetic (e.g., polyester) versus natural (e.g., cotton) fabrics of varying assemblies (e.g., semi-nude or fully clothed) on perceptual responses to exercise (Gavin et al. 2001; Brazaitis et al. 2010; Sperlich et al. 2013; De Sousa et al. 2014).

About 50–60% of our athletes favored the COOL versus CTRL ensemble as it related to their level of perceived comfort, cooling, exercise performance, and training suitability. When asked to qualify their choice, the athletes cited the COOL ensemble as being lighter, thinner, more comfortable, and less compressive. These qualitative descriptors accurately depict the differences in yarn weight and size used to create the two ensembles; and are consistent with the most important clothing attributes discussed by Bishop et al. (2013). Collectively, these findings suggest that garment properties related to comfort, and not cooling, per se, are important during high-intensity exercise. As such, consumers are likely to purchase athletic clothing based on its weight, thickness and perceived comfort, even in the absence of measurable (i.e., objective) improvements in exercise tolerance. Future studies, therefore, should consider the role of perceived clothing comfort on exercise performance in endurance-trained athletes, independent of and in relation to the thermal and comfort properties of the garment.

### Methodological considerations

The purpose of the present study was to evaluate the purported “cooling” properties of a synthetic fabric on the exercise performance of endurance-trained athletes in an indoor environment. As such, tests were carried out under ambient laboratory conditions on a stationary bicycle (i.e., no convection) using a high-intensity constant power output exercise test protocol with participants fully clothed (vs. semi-nude) so as to mimic the environment of an indoor training/sporting facility as well as elicit relatively high sweat rates and marked increases in *T*_eso_ within a relatively short period of time. The net effect of the relatively short exercise duration, however, was that fluid (sweat) loss was ∼0.5% of body mass (representing minor dehydration); the rate of heat storage was modest; and the majority of our participants stopped exercise because of intolerable leg discomfort rather than thermal strain. For these reasons, we cannot preclude the possibility that the COOL ensemble may have resulted in improved exercise tolerance had mild-to-moderate intensity exercise of longer duration been carried out in a hot and/or humid environment where (1) more severe dehydration and rates of heat storage would have been present; and (2) exercise performance would have been more severely limited by thermal strain.

The COOL ensemble was not compared to an ensemble made of a natural fabric, such as cotton. Although cotton has good water-absorbing properties, it also tends to retain water, which hinders moisture transfer and impairs heat dissipation from the skin. Despite our lack of a natural fabric (cotton) control, several previous studies were not able to demonstrate a difference in thermoregulatory responses during exercise in a hot environment (>30°C) while wearing a cotton garment compared to a garment with professed enhanced “cooling properties” (Gavin et al. 2001; Brazaitis et al. 2010; Sperlich et al. 2013; De Sousa et al. 2014). As the COOL ensemble in this study was compared to a synthetic control ensemble specifically chosen by the manufacturer, and not to a 100% cotton ensemble, the purported superior “cooling” properties of the COOL ensemble, as it may relate to the established “cooling” properties of cotton, remains a matter of conjecture.

## Conclusion

The results of this randomized, double-blind, placebo-controlled, cross-over study do not support the hypothesis that wearing a garment composed of a synthetic “cooling” fabric improves exercise performance of trained athletes by enhancing thermoregulatory, cardiometabolic, ventilatory, and perceptual responses to exercise, at least not during high-intensity cycle ergometer exercise under ambient laboratory conditions that mimic the temperate environment of indoor training/sporting facilities.
